# Appropriate use of a dry powder inhaler based on inhalation flow pattern

**DOI:** 10.1186/s40780-017-0076-9

**Published:** 2017-01-18

**Authors:** Tetsuri Kondo, Makoto Hibino, Toshimori Tanigaki, Stanley M. Cassan, Sakurako Tajiri, Kenichro Akazawa

**Affiliations:** 1 Department of Respiratory Medicine, Shonan Fujisawa Tokushukai Hospital, 1-5-1 Kandai, Tsujido, Fujisawa, Kanagawa 251-0041 Japan; 2 Division of Respiratory Medicine, Atsugi Circulation Clinic, 3-5 Izumi-chou, Atsugi, Kanagawa 243-0013 Japan; 3 Department of Medicine, Stanford University, Stanford, CA 94305-2004 USA; 4 Department of Medicine, Tokai University Oiso Hospital, 21-1 Gakkyo, Oiso, Kanagawa 259-0198 Japan

**Keywords:** Dry powder inhaler, Instruction, Inhalation flow profile, Self-training

## Abstract

**Background:**

An optimal inhalation flow pattern is essential for effective use of a dry powder inhaler (DPI). We wondered whether DPI instructors inhale from a DPI with an appropriate pattern, and if not, whether self-training with visual feedback is effective.

**Methods:**

Subjects were 14 pharmacists regularly engaged in instruction in DPI use. A newly designed handy inhalation flow visualizer (Visual Trainer: VT) was used to assess inhalation profiles and to assist in self-training. With a peak inhalation flow rate (PIFR) > 50 L/min, time reaching PIFR (T_PF_) < 0.4 s, inhalation volume (V_I_) > 1 L, and flow at 0.3 s after the onset of inhalation (F_0.3_) > 50 L/min, the pattern was considered optimal.

**Results:**

Using Diskus or Turbuhaler 12 and 10 subjects respectively inhaled with a suitable PIFR. Those with a satisfactory F_0.3_ were 10 and 7 respectively. The T_PF_ was short enough in only 1 and 2 respectively. All 14 subjects inhaled deeply (V_I_) through Diskus, and 10 did so through Turbuhaler. In the self-training session, only 3 subjects satisfied all three variables at the first trial, while 2 or 3 trials were required in other subjects. Among the three variables, optimal T_PF_ was the most difficult to attain. Once a satisfactory inhalation pattern was achieved using one DPI, eleven out of 12 subjects inhaled with a satisfactory pattern through the other DPI.

**Conclusion:**

Visualization of the inhalation flow pattern facilitates the learning of proper inhalation technique through a DPI.

## Background

Inhalation with an optimal flow pattern is mandatory for effective use of dry powder inhalers (DPIs). The ISAM (International Society of Aerosol in Medicine)/ERS (European Respiratory Society) task force encourages inhalation with different flow patterns using reservoir/blister-type or capsule-type DPIs [1]. However, convenient devices depicting inhaled flow pattern are currently unavailable. Concerning this issue, we previously reported a low-cost and handy inhalation profile analyzer [2] which displays a trajectory of the inhaled flow through the DPI, and some parameters such as peak inhaled flow rate (PIFR), time reaching the PIFR (T_PF_), and inhaled volume (V_I_) are also displayed. Using this device named a Visual Trainer, we found that many patients who were currently treated with a DPI did not inhale with a suitable flow pattern [2]. We then wondered whether DPI instructors themselves inhaled with an ideal inhalation pattern since they also were unaware of their inhalation flow profiles through the DPI. Therefore, as the first purpose of the present study using the Visual Trainer, we assessed DPI inhalation profiles of pharmacists regularly engaged in instruction in DPI use. Pharmacists are largely responsible for DPI instruction in Japan. If they did not inhale with an appropriate flow pattern, as the second purpose, we assessed the effectiveness of visual feedback using the Visual Trainer for self-training.

## Methods

**Fig. 1:**
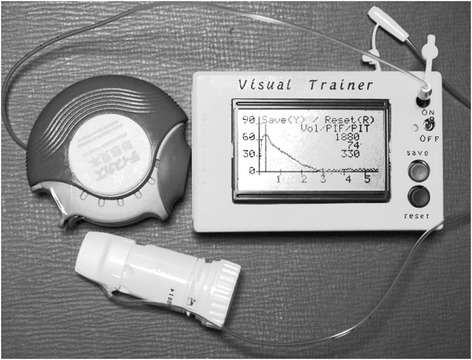
Appearance of the visual trainer

This study was permitted by the Human Ethics Committee of Shonan Fujisawa Tokushukai Hospital (approval number 14–019). This study was registered in University Hospital Medical Information Network-Clinical Trial Registry (April 24, 2013, ID: UMIN 000023136). Figure 1 shows the appearance of the Visual Trainer. Detailed descriptions are shown elsewhere [2]. In brief, it is 12.5 × 8.0 × 3.5 cm in size and 300 g (including batteries) in weight. The cost of its parts is approximately $100. This system continuously measures pressure in the mouthpiece of a DPI (P_aw_) through a fine plastic tube. The P_aw_ signals are converted to digital ones with a 10 bits A to D converter, and then processed by a microcomputer. Sampling rates are each 10 ms for 0–1.27 s, 20 ms for 1.27–2.56 s, and 50 ms for 2.56–5.76 s. Inhalation flow rate is calculated with the equation, flow = constant × P_aw_^0.5^, and is continuously displayed on a GLCD (graphic liquid cell display). The PIFR, T_PF_, and V_I_ are calculated and also displayed on the GLCD. The P_aw_ data can be stored on an SD-card for later analysis.

**Fig. 2:**
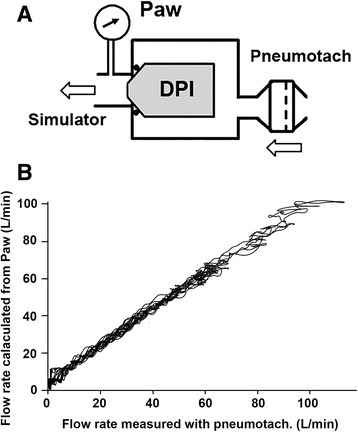
Accuracy of the Visual Trainer. **a**: Inhalation flow was measured simultaneously using both the square root of airway pressure (P_aw_) and a pneumotachometer while the DPI was placed in an airtight box. **b**: Results of 7 consecutive inhalations from Diskus with different flow rates

Before conducting the human study, we confirmed the accuracy of the Visual Trainer. In this experiment, Diskus was placed in an airtight box as shown in Fig. 2a and an inhalation simulator inhaled several times. Pressure in the mouthpiece and airflow at the inlet of the airtight box were continuously measured. Airflow was measured with a pneumotachometer (TV-112 T, Nihon Kohden, Tokyo, Japan).

**Fig. 3:**
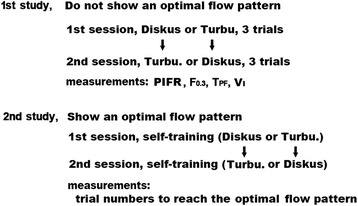
Study protocol

The subjects were 14 pharmacists working in Shonan Fujisawa Tokushukai Hospital. They participated voluntarily in the study after signing an informed consent. All were engaged in DPI instruction to patients using In-checks. Figure 3 shows the protocol for the study. The study consisted of 2 parts. On the first day, the subjects inhaled 3 times from both Diskus (DPI for Adair or Advair) and from Turbuhaler (DPI for Symbicort outside of the US) using depleted devices devoid of active drug, with a flow pattern which they believed to be optimal. When one subject began with Diskus the next subject of the group began with Turbuhaler. In this study, one researcher collected data from all of the subjects using a single Visual Trainer. The recorded data was later analyzed using Microsoft Excel and StatView 5.0 (SAS institute, Australia). Data differences of < 0.05 using a Wilcoxon single-ranked test were considered significant.

The second study was conducted 2 weeks later. Only 13 of the 14 pharmacists participated in the study because one subject had already achieved an acceptable inhalation profile. We described the optimal inspiratory flow pattern proposed by the ISAM/ERS task force [1]. Then, without a training session, twelve subjects began self-training for a proper DPI inhalation using Visual Trainers. They repeated inhalations until reaching all the following 3 parameters; PIFR > 50 L/min, T_PF_ < 0.4 s, V_I_ > 1.0 L. We did not direct which DPI device was to be used initially (1st session). In this study 5 visual trainers were distributed among 13 subjects. This enabled the subjects to complete each trial at intermission between their pharmacy duties.

## Results

Figure 2b shows the results of 7 consecutive inhalations from Diskus with different flow rates with the time to reach the peak inhalation flow at 0.5 s. The abscissa is output from the penumotachmeter and the ordinate is flow rate calculated from airway pressure, ie, output from the Visual Trainer. There was good correlation between the two outputs, confirming the accuracy of the Visual Trainer.

**Fig. 4:**
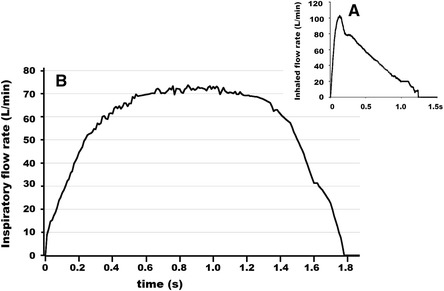
An example of flow trajectory while a subject inhaled through Diskus. **a**: An optimal pattern [2]. **b**: Most frequently observed pattern

There were some inter-subject and inter-trial variations in inhaled flow patterns. Among them, a pattern shown in Fig. 4 was most frequently observed. This pattern was recorded while the subject inhaled through Diskus, and a similar pattern was observed in this subject while inhaling through Turbuhaler. This pattern was classified as trapezoid in the previous study [3]. It was characterized by the following variables; PIFR 77.5 L/min, T_PF_ 0.76 s, V_I_ 1.57 L and F_0.3_ 57.6 L/min, suggesting that the subject inhaled deeply and strongly with rapid rise in flow.

**Fig. 5:**
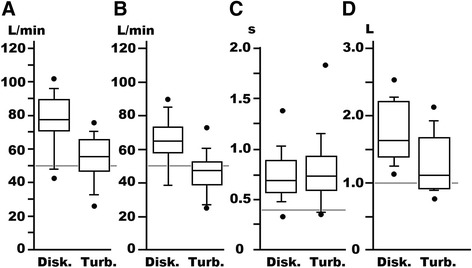
PIFR (**a**), F_0.3_ (**b**), T_PF_ (**c**), and V_I_ (**d**) of individual subjects in the first study. Gray lines indicate threshold values for individual parameters

Figure 5 shows PIFR, F_0.3_, T_PF_, and V_I_ of individual subjects in the first study. The gray lines represent thresholds for each parameter. Their validity will be described later. It can be seen that many subjects inhaled forcefully (PIFR) and deeply (V_I_). In many subjects, peak flow did not appear during early inhalation (T_PF_), but F_0.3_ was close to PIFR. Exact values and statistical significance of Fig. 4 are listed in Table 1. It will be recognized that the subjects inhaled more deeply (V_I_), more rapidly (F_0.3_), and with significantly more strength (PIFR),using Diskus compared with Turbuhaler. T_PF_ in the two groups tended to be long and no significant difference was found. Using Diskus 12 subjects inhaled with a suitable PIFR as did 10 using Turbuhaler. All 14 subjects inhaled deeply (V_I_ >1.0 L) using Diskus and 10 did so using Turbuhaler. The time to reach PIFR was less than 0.4 s in only 1 subject (Diskus) and 2 subjects (Turbuhaler). Flow rate at the early phase of inhalation (F_0.3_) was satisfactory in 10 subjects with Diskus use, and 7 subjects with Turbuhaler use. There was only one subject who reached all the thresholds in both Diskus and Turbuhaler trials.

**Table 1:** Inhalation parameters before the self-trainings (median, 75th and 25th percentiles)

	Diskus	Turbuhaler	Difference
PIFR (L/min)	77.6, 89.3, 72.2	55.3, 64.5, 47.8	significant
V_I_ (L)	1.63, 2.19, 1.40	1.11, 1.66, 0.95	significant
F_0.3_ (L/min)	64.9, 71.6, 58.3	47.2, 52.1, 39.1	significant
T_PF_ (s)	0.69, 0.89, 0.57	0.74, 0.93, 0.61	ns

**Fig. 6:**
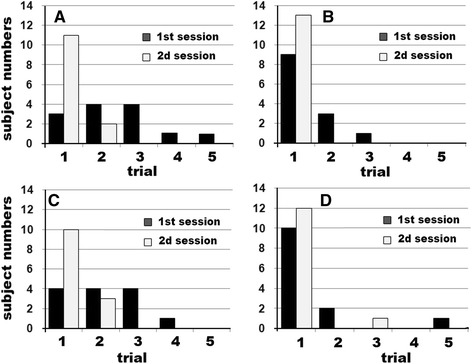
Number of subjects who fulfilled the criteria at each trial. **a**; subject number who achieved all three criteria. **b**; those who reached PIFR threshold. **c**; those who reached T_PF_ threshold. **d**; those who reached V_I_ threshold. In the 1st session, subjects started the inhalation trial with either Diskus or Turbuhaler. In the 2nd session they selected the other DPI

In the second study, all the subjects fulfilled the criteria after a few self-training attempts. Figure 6 shows the number of training trials needed for subjects to fulfill the criteria for the optimal inhalation pattern. Since T_PF_s decreased remarkably after self-training, i.e., median T_PF_ 0.34 s for Diskus and 0.31 s for Turbuhaler, F_0.3_s were almost the same as PIFR, and thus F_0.3_s are not shown in Fig. 6. Ten subjects chose Diskus in the first session, and the remaining subjects chose Turbuhaler first. Panel A shows subject numbers who fulfilled all three criteria at each trial. In using the initially selected DPI (1st session), only 3 subjects fulfilled all three criteria at the first trial. In most subjects 2 or 3 trials were necessary to fulfill the criteria. In contrast, in the training using the second selected DPI (2nd session), eleven out of 12 subjects reached the three thresholds on the first trial. Panel B-D show trial numbers to achieve individual parameters. In panels B and D, it can be seen that most of the subjects inhaled with a suitable PIFR and V_I_ at the first trial of either the first or second session. In contrast, an adequate T_PF_ was difficult to attain at the first study of the first session while many subjects attained this parameter at the first trial of the second session. Further comparison between those who chose Diskus first and Turbhaler first was not possible because most of the subjects chose Diskus first. However no trends were apparent between them.

## Discussion

Inhalation therapy using DPIs is now the mainstay of treatment of COPD and bronchial asthma. Since drug dispersion and generation of fine particles are driven by energy from inhaled flow through the DPI, inhalation flow pattern including flow rate and timing of peak flow impact its efficiency. Once dispersed from the DPI, powdered drugs are propelled through the airways and then precipitate in the large and small bronchi making strength and depth of inhalation important. However, compared with instruction in employing a DPI, systematic instruction in inhalation flow through a DPI is not widely practiced. One reason may be poor recognition of DPI-specific inhalation patterns [1] by many DPI instructors, and this may be partly due to lack of a convenient device to visualize inhalation flow pattern. In-check and inhalation trainers currently used are not satisfactory for this purpose because these devices depict inhalation flow rate at only one point in time. A few systems visualizing the time course of inhalation flow rate from a DPI have been reported [4–6]. However, all of these systems are either complicated or expensive, and thus are not suited to use in clinical practice. Visual trainer, a low-cost and handy inhalation profile analyzer, potentially solves these problems.

### Threshold determination

Concerning the threshold value, we used a PIFR > 50 L/min because this value is recommended in use of medium/high-resistance DPIs [1]. According to one study on children well trained in DPI use [7], in which the peak of drug dispersion from Diskus was 0.16 ± 0.14 s (mean ± SD) and that from Turbuhaler was 0.19 ± 0.03 s, the PIFR should appear at around 0.16–0.19 s after onset of inhalation. In our previous study using the inhalation trainers in healthy adults, the T_PF_ through Diskus was 0.44 ± 0.17 s and that through Turbuhaler was 0.53 ± 0.23 s [8]. Thus, we set the requirement of T_PF_ as < 0.4 s. Although the optimal V_I_ from a DPI is not established, 80% vital capacity is recommended in pMDI (pressurized metered dose inhaler) use. However, favorable pulmonary drug deposition (comparisons of 20, 50 and 80% vital capacities) [9] or drug absorption (functional residual volume vs. total lung capacity) [10] was reported with smaller inhalation volume. We set 1.0 L as a minimum requirement for V_I_. Inhaled flows at 0.3 s after onset of inhalation (F_0.3_), which represents the flow rate at termination of drug dispersion [7], was also measured.

The ISAM/ERS task force recommends a rapid and forceful inhalation for reservoir or blister-type DPI [1]. Once drug has been dispersed from a DPI, inhalation flow rate should be low to avoid precipitation in the upper airway. Studies on pMDI's suggest that a suitable flow after drug dispersion is approximately 30 L/min [11]. Thus, practically the best inhalation pattern for Diskus and Turbuhaler may be that shown as Fig. 4a [2], and this pattern is exactly the same as that proposed by the ISAM/ERS task force.

### Pharmacists’ inhaled pattern

As shown in Figs. 5a and d, most of the pharmacists inhaled forcefully (high PIFR) and deeply (large V_I_) through both DPIs. The steepness of inhaled flow was assessed by two parameters, T_PF_ and F_0.3_. Most of the subjects inhaled with an unsatisfactory T_PF_ through either DPI (Fig. 5c). Although T_PF_ is a reasonable parameter of flow steepness it is not a suitable index in evaluation of the trapezoid pattern (see Fig. 4). Since the trapezoid pattern was frequently seen in this study, we adopted F_0.3_ as an additional parameter of flow steepness. A satisfactory F_0.3_ was observed in 12 of 14 subjects in Diskus use but in only half of the subjects with Turbuhaler use (Fig. 5b). Even though high F_0.3_ is achieved, the trapezoid pattern is not preferable because protracted high inhaled flow may adversely affect both drug delivery and precipitation in the pulmonary airways. Therefore, we concluded that our subjects, who are regularly engaged in DPI inhalation instruction, did not themselves inhale with an appropriate flow pattern.

### Effects of training with visual feedback

Steep increase in inhalation flow has a marked effect on drug dispersion [12] as well as fine particle generation from a DPI [13]. Unfortunately, many of our subjects failed to achieve sufficiently rapid inhalation in terms of T_PF_ and F_0.3_. This finding is not surprising because currently available devices such as In-Check or trainer whistles do not show flow trajectory. Al-Showair et al. [14] have reported that an optimal inhalational flow pattern was not achieved following verbal instruction, and we also confirmed this [3]. Furthermore, even after describing an optimal flow pattern, only 20% of the subjects in the present study achieved a satisfactory pattern at the first trial. Thus, currently available techniques including verbal instructions, flow trainer devices, and description of optimal flow are limited. In contrast, after self-training with Visual Trainer T_PF_s decreased remarkably. Since drug dispersion occurs at very early inhalation, early development of PIFR augments inhalation efficiency. As has been reported, some maneuvers augment inhalation depth and strength [15] but no strategy for achieving rapid inhalation has been proposed. Although our study had no control group, the results suggest that self-training with visual feedback is a strong tool to resolve this problem.

Once a subject had achieved an optimal flow pattern using one DPI, many subjects inhaled optimally through the other DPI (Fig. 6a). This occurred whether the DPIs were changed from those with medium/high to low resistance or vice versa. This suggests that, when the type of DPI is changed, detailed instruction in flow pattern is not required in well-trained patients.

### Usefulness of visual trainer

With regard to the Visual Trainer in this study, we were able to use 5 devices concurrently in the 2nd trial owing to their cheap and handy attributes. This enabled the collection of data in only one day while all the pharmacists were engaged in their hospital duties. Short-term data collection might also preclude information exchange among the subjects who, in the self- training study, could have impacted the results. We have reported that patients regularly using DPIs do not always inhale with adequate flow patterns [2].The present results portend the effectiveness of Visual Trainer in training such patients.

## Conclusion

Visualization of the inhalation flow pattern facilitates the learning of proper inhalation technique through a DPI. An optimal inhalation pattern can be easily achieved by self-training when the inhalation flow pattern is displayed.

## Abbreviations

COPD: Chronic obstructive pulmonary disease
DPI: Dry powder inhaler
ERS: European respiratory society
F_0.3_: Inhaled flows at 0.3 s after onset of inhalation
GLCD: Graphic liquid cell display
ISAM: International society of aerosol in medicine
P_aw_: Pressure in the mouthpiece of a DPI
PIFR: Peak inhaled flow rate
pMDI: Pressurized metered dose inhaler
T_PF_: Time to reach the PIFR
V_I_: Inhaled volume.
